# Vaccine efficacy induced by virus-like particles containing *Leishmania donovani* surface glycoprotein GP63

**DOI:** 10.1371/journal.pntd.0012229

**Published:** 2024-06-10

**Authors:** Keon-Woong Yoon, Ki Back Chu, Gi-Deok Eom, Jie Mao, Fu-Shi Quan

**Affiliations:** 1 Department of Biomedical Science, Graduate School, Kyung Hee University, Seoul, Republic of Korea; 2 Department of Parasitology, Inje University College of Medicine, Busan, Republic of Korea; 3 Department of Infectious Disease and Malaria, Paik Institute of Clinical Research, Inje University, Busan, Republic of Korea; 4 Medical Research Center for Bioreaction to Reactive Oxygen Species and Biomedical Science Institute, Core Research Institute (CRI), Kyung Hee University, Seoul, Republic of Korea; 5 Department of Medical Zoology, School of Medicine, Kyung Hee University, Seoul, Republic of Korea; Bose Institute, INDIA

## Abstract

*Leishmania donovani* surface glycoprotein 63 (GP63) is a major virulence factor involved in parasite escape and immune evasion. In this study, we generated virus-like particles (VLPs) expressing *L*. *donovani* GP63 using the baculovirus expression system. Mice were intramuscularly immunized with GP63-VLPs and challenged with *L*. *donovani* promastigotes. GP63-VLP immunization elicited higher levels of *L*. *donovani* antigen-specific serum antibodies and enhanced splenic B cell, germinal center B cell, CD4^+^, and CD8^+^ T cell responses compared to unimmunized controls. GP63-VLPs inhibited the influx of pro-inflammatory cytokines IFN-γ and IL-6 in the livers, as well as thwarting the development of splenomegaly in immunized mice. Upon *L*. *donovani* challenge infection, a drastic reduction in splenic parasite burden was observed in VLP-immunized mice. These results indicate that GP63-VLPs immunization conferred protection against *L*. *donovani* challenge infection by inducing humoral and cellular immunity in mice.

## Author summary

Visceral leishmaniasis is a neglected tropical disease that can be fatal in humans if left untreated. Unfortunately, despite the urgent need, an efficacious vaccine for this parasitic disease continues to remain under development. Though challenging, we attempted to develop an experimental leishmaniasis vaccine using the highly immunogenic virus-like particle (VLP) platform that expressed the glycoprotein 63 antigen of *Leishmania donovani*, a causative agent of visceral leishmaniasis. We used this vaccine to immunize mice and after two immunizations, mice were challenge infected with *L*. *donovani* to assess vaccine-induced protection. Our results revealed that compared to the unimmunized control mice, mice immunized with the experimental vaccines were well-protected against developing visceral leishmaniasis. Specifically, VLP vaccine immunization ensured that immune responses necessary for protection were induced to high levels and this contributed to suppressing leishmaniasis-associated pathologies as well as the parasite propagation in mice. Overall, we demonstrated that this VLP-based vaccine can elicit adequate protection against visceral leishmaniasis and propose further research to enhance and optimize its efficacy.

## Introduction

*Leishmania donovani* is a digenetic parasite whose life cycle involves a vertebrate host and the sand fly. This organism is responsible for causing severe visceral leishmaniasis (VL) in humans and post-kala-azar dermal leishmaniasis development was reported to be almost exclusively associated with *L*. *donovani* infection [1,2]. While the global incidence rate for this fatal disease has been decreasing, VL is still prevalent in the Indian subcontinent as well as in parts of Africa and South America [2]. Efficacious vaccines for human VL remain unavailable to date, but patients who convalesced from VL were reported to be immune to subsequent infection which supports the rationale for VL vaccine development [3]. To date, the immunogenicity of various *Leishmania* spp. antigens were evaluated. One potential candidate antigen for VL vaccines could be the zinc metalloproteinase GP63, a 63 kDa protein found on the surface of promastigotes that aids in parasite propagation [4]. Given that GP63 is also a virulence factor involved in the suppression of the host’s immune response which involves impaired macrophage functioning [5,6], raising immune responses directed at this antigen could contribute to effectively inhibiting the parasite’s survival.

Several approaches to developing a VL vaccine using the *L*. *donovani* GP63 antigen have been ongoing for decades using murine models. Yet, considerable differences in protective efficacies were reported in these earlier VL vaccine studies. Encapsulating GP63 antigen in cationic liposomes and inoculating these emulsified antigen components into BALB/c mice conferred partial protection against experimental visceral leishmaniasis challenge infection [7,8]. Immunogenicity of the GP63 was further enhanced when the antigen was encapsulated in monophosphoryl lipid A-trehalose dicorynomycolate in comparison to cationic liposome entrapment [9]. Four GP63 T cell epitopes were predicted using bioinformatics tools, but their immunization resulted in marginal induction of immune responses in volunteers [10]. A multi-antigenic DNA vaccine encoding GP63 and several other antigens failed to induce adequate protection against *L*. *infantum* challenge infection in canines [11]. Combinatorial protein vaccination involving GP63 and Hsp70 effectively reduced parasite load and enhanced Th1 cytokine production for several months and its efficacy could be further enhanced by supplementing monophosphoryl lipid A [12,13]. Heterologous immunization strategies were also demonstrated to be effective. Alternating between DNA and protein vaccines expressing the *L*. *donovani* GP63 antigen conferred protection in BALB/c mice. This approach significantly reduced the parasite burden upon *L*. *donovani* infection even partially inhibited footpad swelling in *L*. *major*-challenged mice, signifying room for further development [14]. In one comparative study involving amastin, Kmp-11, and GP63, DNA prime-protein boost immunizations using vaccines co-expressing GP63 and Kmp-11 elicited the best protection in BALB/c mice [15,16].

While these earlier GP63 vaccine studies paved the path to its further development, none have attempted to incorporate GP63 as a vaccine antigen using the highly immunogenic virus-like particle (VLP) vaccine platform. VLPs can address several limitations associated with the aforementioned vaccine platforms. For instance, DNA vaccines are frequently inoculated at high doses to ensure successful plasmid delivery into the cell nucleus but even then, these vaccines tend to elicit low immunogenic profiles [17]. There are also safety issues, as exemplified by an earlier report documenting anti-DNA antibody-mediated autoimmune disease [18]. Protein subunit vaccines are safe but this comes at the expense of poor immunogenicity, thereby mandating adjuvant incorporation and multiple immunizations [19]. As a safe and highly immunogenic vaccine platform, expressing the GP63 antigen on VLPs could aid in future VL vaccine development. Several VLP-based VL vaccine studies have been conducted to date, including a multivalent vaccine comprising leishmanial and sand fly salivary antigens, as well as a Qβ phage-derived VLP vaccine displaying the carbohydrate α-Gal conjugate as antigens, both of which conferred protection in mice [20,21]. We also demonstrated that baculovirus-derived VLPs expressing the *L*. *donovani* promastigote surface antigen (PSA-VLP) were efficacious in mice [22]. However, our previous study was solely focused on the protection contributed by VLP-induced humoral immunity. To confirm that VLPs also elicit T cell responses required for parasite clearance, as well as to improve the efficacy of our VLP vaccination approach, we expressed the immunogenic *L*. *donovani* GP63 antigen on the surface of influenza M1 protein and evaluated immune parameters contributing to protection in mice. Our findings provide encouraging results for further application of VLPs for VL vaccines.

## Materials and methods

### Animal ethics and handling

Six-week-old female BALB/c mice, each weighing between 17~18g, were purchased from NARA Biotech (Seoul, South Korea). Mice were housed in a specific pathogen-free animal facility with easy access to food and water at Kyung Hee University. To minimize any distress to the animals, all essential precautions were taken. Humane intervention point was determined as weight loss exceeding 20% of their initial value and mice meeting this criterion were immediately euthanized with CO_2_. All animal experiment procedures were approved by the Institutional Animal Care and Use Committee (IACUC) of Kyung Hee University (permit number KHUASP (SE) 21–250).

### *L*. *donovani* propagation and viability assay

*L*. *donovani* strain Ld1S (MHOM/SD/62/1S-CL2D) was initially maintained in BALB/c mice. After promastigote isolation from the spleen, they were cultured in T25 flasks (SPL Life Sciences, Pocheon, South Korea) at 27°C with modified M199 media supplemented with 10% heat-inactivated fetal bovine serum, 1% penicillin/streptomycin, L-glutamine, 25 mM HEPES, 0.1 mM adenine, 0.0005% hemin, 1% folic acid, 0.0001% biotin, 0.0001% biopterin, and 4.62 mM NaHCO_3_ (Sigma-Aldrich, St. Louis, MO, USA) as previously described [23]. For *in vitro* cultivation, *L*. *donovani* promastigotes were seeded at an initial density of 1x10^6^ parasites per T25 flask. Observations indicated a noticeable increase in parasite growth starting on day 3, with a subsequent decline occurring on day 9. Live and dead promastigotes were differentiated based on fluorescence emission and live promastigotes were counted from 9 different fields of view.

### Characterizing the GP-63 VLPs

VLPs were characterized using the methods previously described [24]. The initial step involved confirming the presence of a transmembrane (TM) domain within the GP63 gene. This was achieved through *in silico* analysis, where GP63 gene sequences were scrutinized. Phobius software was used for TM domain site prediction. VLP protein concentrations were quantified using the QuantiPro BCA Assay kit (Sigma-Aldrich, St. Louis, MO, USA). For western blot, VLP samples were initially resolved via SDS-PAGE and subsequently transferred to a nitrocellulose membrane at 70V for 90 minutes. Membranes were blocked with 5% skim milk prepared in Tris-buffered saline with 0.1% Tween-20 (TBST) at RT for 1 h. After incubating the membrane overnight at 4°C with sera of *L*. *donovani*-infected mice collected at 10 weeks post-infection (wpi) (1:200 dilution in TBST), membranes were washed and incubated with horseradish peroxidase (HRP)-conjugated anti-mouse IgG for 1 h at RT. Protein bands were visualized using a ChemiDoc system from Bio-Rad, Hercules, CA, USA, following exposure to enhanced chemiluminescence (ECL). To visualize the structural morphology of the assembled VLPs, transmission electron microscopy (TEM) was performed. Briefly, VLPs were adsorbed onto copper grids and stained with uranyl acetate. Images were acquired using a high-voltage electron microscope system (JEM-1400 Plus at 120 kV and the JEM-1000BEF at 1000 kV, JEOL Ltd., Tokyo, Japan). For the antibody dilution experiment, enzyme-linked immunosorbent assay (ELISA) was performed. Immunoplates were coated with GP63-VLPs at a concentration of 2 μg/mL in carbonate coating buffer, overnight, 4°C. Wells were blocked with 0.2% gelatin prepared in 0.1 M PBS with 0.05% Tween-20 (PBST). *L*. *donovani* infection sera or monoclonal GP63 antibodies were used as primary antibodies and these were inoculated into respective wells (1:100 dilution). Plates were incubated at 37°C for 1 h, and subsequently incubated with HRP-conjugated goat anti-mouse IgG secondary antibody (1:2,000 dilution in PBS) for 1 h at 37°C. Color development was achieved by dissolving o-phenylenediamine in citrate substrate buffer containing 0.03% H_2_O_2_. Optical density readings at 490 nm were measured using a microplate reader (Enzo Life Sciences, Farmingdale, NY, USA).

### Immunization and challenge infection protocol in mice

A total of 32 mice were subdivided into 4 groups (n = 8 per group): unimmunized (Naïve), unimmunized infection control (Naïve+Cha), immunized with 10 μg of GP63-VLPs, and immunized with 50 μg of GP63-VLPs. The immunization procedure entailed two intramuscular injections of GP63-VLPs with respective doses at 4-week intervals. Following the boost immunization, mice were challenged with 1 x 10^8^ *L*. *donovani* promastigotes through the retro-orbital route as previously described [25]. On week 18, which corresponds to 10 wpi, all of the mice involved in the study were euthanized to facilitate organ sampling and perform *ex vivo* immunological assays. The entire animal experiment was performed once, with each immunological assay being performed three times using individually processed samples. Assays were performed with technical replicates.

### Cloning of the *L*. *donovani* GP63 antigen

Successful pFastBac clonal vector expressing the codon-optimized *L*. *donovani* GP63 (accession no. AJ495002.1; 1,926bp) was purchased from GenScript (Piscataway, NJ, USA). The recombinant GP63-pFastBac plasmids were transformed into DH5α competent cells. To verify the expression of the GP63 gene in these colonies, restriction enzyme digestion using BamHI and HindIII was performed, which was immediately followed by sequencing analysis. Once GP63 expression was confirmed, clones were further transformed into DH10Bac competent cells. From these cells, bacmid DNA was extracted using the QIAprep Spin Miniprep kit (Qiagen, Venlo, Netherlands).

### Production and assembly of recombinant baculovirus and virus-like particles expressing the GP63 gene

The process of producing recombinant baculoviruses (rBVs) expressing the GP63 gene was carried out following the manufacturer’s instruction (Bac-to-Bac Expression System, Thermo Fisher Scientific, Waltham, MA, USA). The bacmid DNA, obtained from successful DH10Bac transformants, was transfected into Sf9 cells using Cellfectin II reagent (Invitrogen, Carlsbad, CA, USA). Supernatants containing the GP63-rBVs were meticulously collected. For VLP assembly, Sf9 cells were co-infected with GP63-rBVs and influenza M1-rBVs. At 4 days post-infection, supernatants were collected and cellular debris was carefully removed via centrifugation at 6,000 rpm, 30 min, 4°C. The supernatant fraction containing the GP63-VLPs was concentrated through ultracentrifugation at 30,000 rpm, 30 min, 4°C. GP63-VLPs were purified through 15-30-60% sucrose gradients. After centrifugation at 30,000 rpm for 1 hour at 4°C, the VLP pellets were resuspended in 0.1 M PBS and stored at 4°C overnight. The concentration of GP63-VLPs was determined using the QuantiPro BCA Assay kit (Sigma-Aldrich, St. Louis, MO, USA).

### Sample preparation for immunological assays

Blood samples were collected from the mice via retro-orbital plexus puncture 1 week after each immunization. Sera were collected by centrifuging the blood samples at 5,000 rpm for 10 minutes. The spleens and livers of the mice were processed at week 18. Spleens were homogenized, and the resulting homogenates were centrifuged at 1,000 rpm for 5 minutes. The supernatants obtained were stored at -80°C for subsequent use in limiting dilution assays (LDA). Red blood cells were removed from the pelleted cell fraction using RBC lysis buffer (Sigma-Aldrich, St. Louis, MO, USA) and cells were washed twice with PBS. Single cell suspensions of splenocytes were subsequently used for flow cytometry assays. Liver tissues were homogenized in 1 ml of cold PBS and centrifuged at 6,000 rpm for 10 minutes at 4°C. The supernatants from liver homogenates were harvested and stored at -80°C for cytokine assays.

### Detection of *L*. *donovani*-specific antibody response

The detection of *L*. *donovani*-specific IgG levels was evaluated using ELISA [22]. Briefly, immunoplates were coated with either sonicated *L*. *donovani* promastigotes at a concentration of 5 μg/mL or GP63-VLP at 2 μg/mL in carbonate coating buffer overnight at 4°C. The next day, wells were blocked with 0.2% gelatin prepared in PBST for 1 h at 37°C. Diluted sera from each group (1:50 dilution in PBS) were used as primary antibodies. Plates were incubated at 37°C for 1 hour. HRP-conjugated goat anti-mouse IgG antibody (1:2,000 dilution in PBS) was added to each well and incubated for 1 hour, 37°C. O-phenylenediamine dissolved in citrate buffer with 0.03% H_2_O_2_ was used as substrate and reactions were stopped with 2N H_2_SO_4_. Optical density readings at 490 nm were measured using a microplate reader (Enzo Life Sciences, Farmingdale, NY, USA).

### Flow cytometric analysis of splenic immune cell populations

Splenocytes were prepared for flow cytometry as previously described [22]. For each mouse, 1 x 10^6^ splenocytes were isolated and then stimulated with 5 μg/mL of sonicated *L*. *donovani* promastigote antigen at 37°C for 2 h with 5% CO_2_. Cells were subsequently stained with respective surface markers to detect *L*. *donovani*-specific CD4^+^ T cells, CD8^+^ T cells, B cells, and germinal center B (GC B) cells. All of the fluorophore-conjugated antibodies were purchased from BD Bioscience (Franklin Lakes, NJ, USA) and Thermo Fisher Scientific (Waltham, MA, USA) and are as follows: CD3 (FITC), CD4 (PE-Cy7), CD8 (PE), CD19 (PE-Cy7), IgD (PE), GL7 (PE), B220 (FITC) and B220 (PE). Stained cells were acquired using the Accuri C6 flow cytometer and populations were analyzed with C6 Accuri software (BD Biosciences, Franklin Lakes, NJ, USA).

### Inflammatory cytokine analysis

The livers from mice were homogenized individually and the supernatants were stored as described previously [22]. The levels of pro-inflammatory cytokines, specifically IFN-γ and IL-6, in these hepatic samples were quantified using BD OptEIA ELISA kits (BD Biosciences, Franklin Lakes, NJ, USA). The experiments were conducted following the manufacturer’s guidelines. Cytokine concentrations were then determined by referencing the standard curve generated during the assay.

### Parasite burden estimation

On week 18, mice were euthanized for spleen collection. To assess splenomegaly, the dimensions of each spleen (length x width x height), were individually measured and weighed prior to homogenization. Spleens were homogenized in cold PBS using frosted slide glasses. After centrifugation at 1,500 rpm for 5 minutes, the supernatants were collected, and the fractions containing parasites were further processed. This involved washing with PBS and subsequent centrifugation at 2,000 rpm. The sedimented parasites were then isolated using a Percoll density gradient and, after centrifugation at 6,000 rpm for 45 minutes at 15°C, a distinct clear band was harvested. These samples were washed three times with PBS, resuspended in PBS, and used for limiting dilution assay (LDA). For LDA, flat-bottom 96-well plates containing 180 μL of modified M199 culture media were prepared. The purified parasites were diluted (1:1,000), and 20 μL of this dilution was added to the plates for serial 10-fold dilution. After 4 days of incubation at 27°C, the transformation of amastigotes into promastigotes was confirmed via microscopy. The parasite burden was quantified using the final dilution at which parasites were detected, as previously described [26]. Promastigotes were counted three times from multiple fields of view under a Leica DMi8 microscope (Leica, Wetzlar, Germany).

### Statistical analysis

All data were analyzed using GraphPad Prism version 8 (San Diego, CA, USA). Experiments were performed using individual samples with technical triplicates and are expressed as mean ± SD. One-way ANOVA with Tukey’s *post hoc* test was utilized to determine statistical significance between groups. Statistical significance was denoted using asterisks (*p < 0.05, **p < 0.01, and ***p < 0.001).

## Results

### Animal experimental schedule

**Fig 1:**
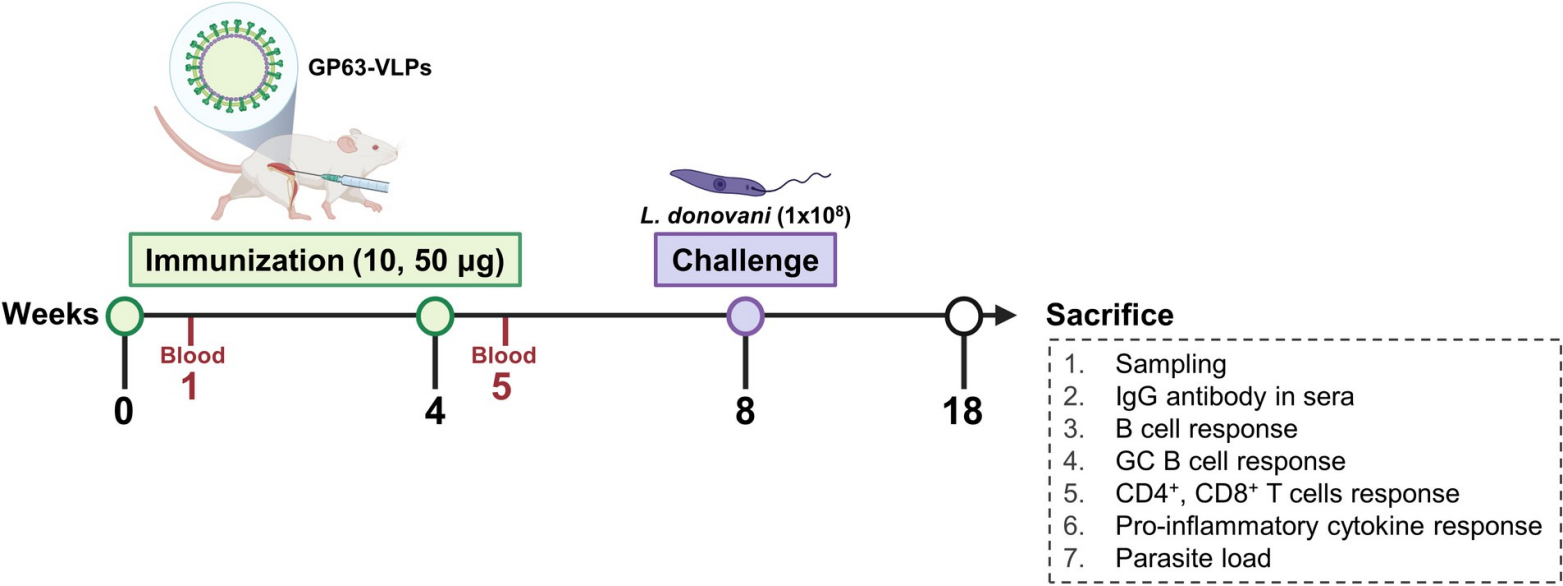
Schematic diagram showing experimental schedule and immunization regimen. BALB/c mice were immunized twice at 4-week intervals, and blood samples were collected from individual mice 1 week after each immunization. Mice were challenge-infected with 1 x 10^8^ *L*. *donovani* promastigotes four weeks after the second immunization. All animals were humanely sacrificed 10 weeks post-infection and immunological assays were performed to evaluate vaccine-induced protection. Created with BioRender.com.

Animal experiments were conducted precisely as depicted in the schematic diagram (Fig 1). In brief, individual BALB/c mice were subjected to intramuscular immunization using VLP vaccines. The doses administered were either 10 or 50 μg, and the immunizations were spaced at 4-week intervals. Four weeks after the second immunization, mice were infected with *L*. *donovani*. The endpoint for these in vivo studies was set at 10 wpi. Upon reaching this endpoint, samples were collected from all four groups of mice. Each sample was then meticulously processed for a series of *ex vivo* analyses. These analyses included flow cytometry to assess immune cell populations, cytokine assays for measuring inflammatory responses, and quantitative assessments of parasite burden. This comprehensive approach allowed for a thorough evaluation of the immune response elicited by the VLP vaccines, as well as the effects of *L*. *donovani* infection in the immunized mice.

### Characterization of vaccines

**Fig 2:**
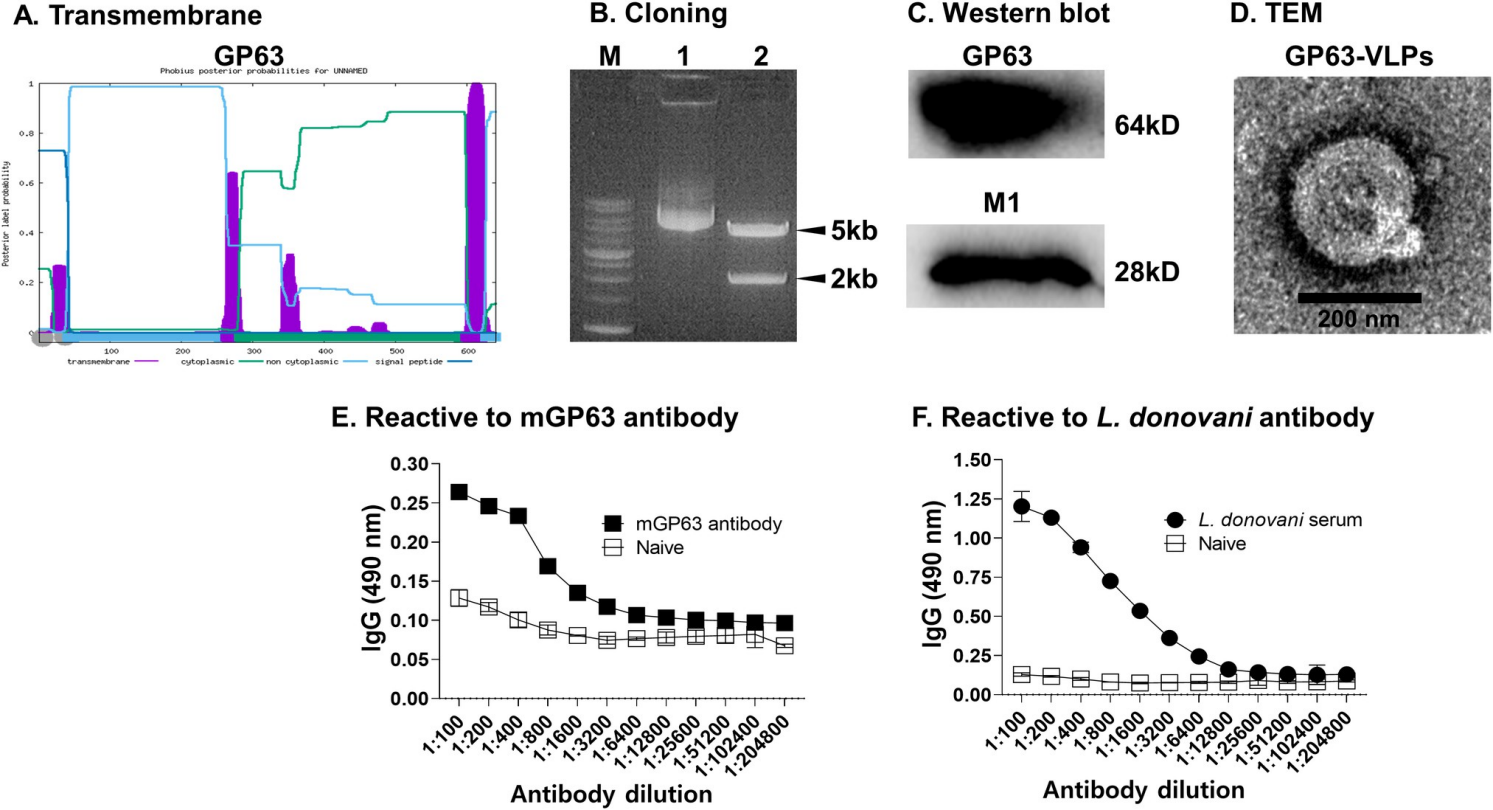
Characterization of the GP63-VLP vaccines. *In silico* transmembrane domain analysis was performed using the Phobius software to confirm the presence of the TM domain within the *L*. *donovani* GP63 (A). DNA from successful clones were harvested and subjected to restriction digestion analysis and visualized using gel electrophoresis (B). GP63-VLPs were resolved on an SDS-PAGE gel and expressions of the influenza M1 and *L*. *donovani* GP63 antigens were confirmed by western blotting (C). The structural morphology of the GP63-VLPs was visualized under transmembrane electron microscopy (D). ELISA was performed and antigen-specific antibody responses were determined using monoclonal anti-GP63 antibody (E) and *L. donovani* infection sera from mice (F).

The presence of transmembrane domains in GP63, which are crucial for VLP assembly, was predicted *in silico* by the web-based program Phobius (Fig 2A). To confirm the successful cloning of the *L*. *donovani* GP63 gene into the pFastBac vector, DNA from transformants was acquired for restriction enzyme digestion (Fig 2B). DNA bands were observed near the 5 kb and 2 kb markers, which correspond to the linearized pFastBac vector and the cloned GP63 gene, respectively. Key protein components of the GP63-VLPs were characterized by western blot analysis (Fig 2C). The GP63 and influenza M1 antigens were detected at 60 kDa and 28 kDa, respectively. The successful assembly of VLPs and their morphologies were visualized by TEM (Fig 2D). GP63-VLPs were coated on immunoplates, and ELISA was performed as described, using serially diluted monoclonal GP63 antibody (Fig 2E) or mouse polyclonal *L*. *donovani* antibody (Fig 2F).

### Growth kinetics and viability assessment of *L*. *donovani* promastigotes

**Fig 3:**
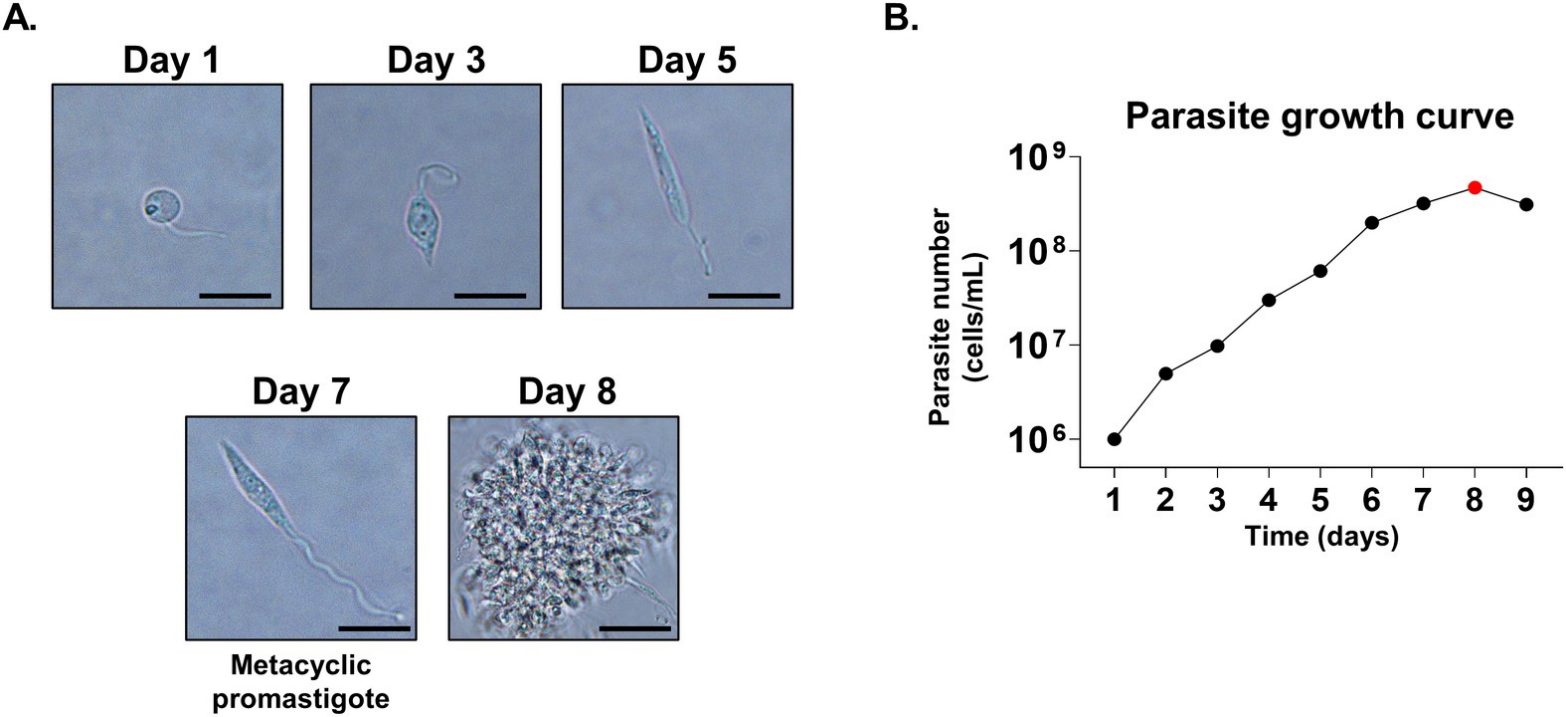
*In vitro* culture of *L*. *donovani* promastigotes. Parasite cultures were monitored daily. Formation of flagella and subsequent proliferation of the parasite was observed under the microscope (A). Parasite growth was determined by counting the motile parasites using a hemocytometer over the span of 9 days (B). Scale bar = 10 μm.

To identify the best stationary phase for parasite growth, 1x10^6^ parasites were cultivated in T25 flasks. Their growth was closely observed on a daily basis, with both the counts and phenotypes of the parasites being systematically recorded each day. On days 1 to 3, the proliferation was barely noticeable, with parasites morphologically resembling procyclic promastigotes. From days 5 to 6, an increased number of parasites were visible under the microscope, exhibiting the morphology of elongated nectomonads. The highest growth, along with the most motile metacyclic promastigote forms, were observed on days 7 and 8. Starting from day 9, motility decreased, accompanied by the suspension and aggregation of dead parasites (Fig 3A). To visualize the point of maximum growth, a growth curve was plotted based on the parasite count data (Fig 3B).

### IgG antibody responses in serum

**Fig 4:**
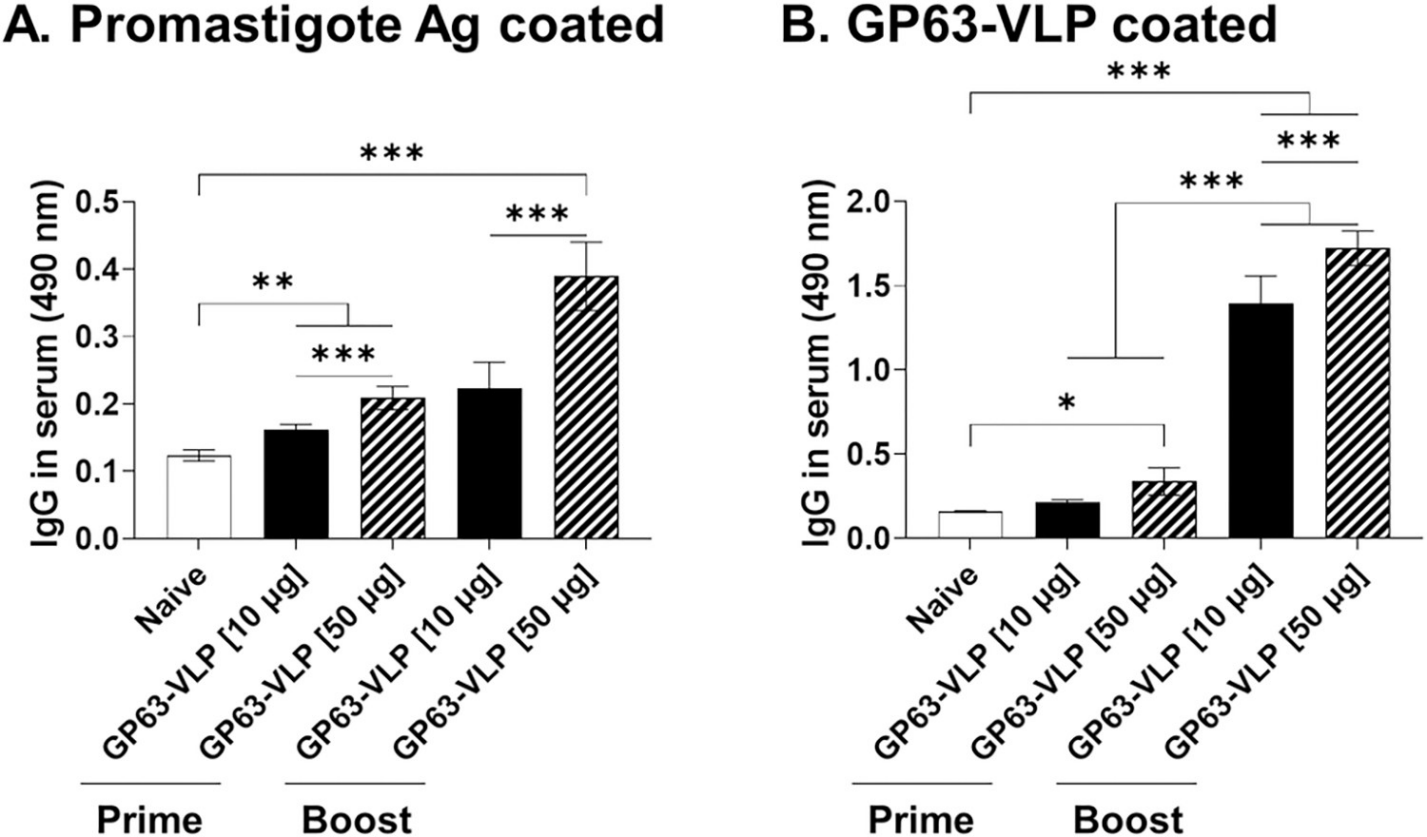
*L*. *donovani* GP63-specific serum antibody responses. Sera were collected 1 week after each immunization to assess antigen-specific antibody responses. Antibodies acquired after prime and boost immunizations were reacted with sonicated *L*. *donovani* promastigote lysates (A) and GP63-VLPs (B). Data are presented as mean ± SD, using individually processed samples (n = 8 per group). Statistical significance between group mean values is indicated by asterisks (*P < 0.05, **P < 0.01, ***P < 0.001).

Serum samples were periodically collected from vaccinated mice to assess their immune response to *L*. *donovani*, focusing specifically on IgG antibody levels. Immunization dose was positively correlated with antibody response induction, irrespective of the antigen used. After the initial vaccination with a 10 μg dose of GP63-VLP, mice showed minimal production of parasite-specific IgG antibodies. Upon boost immunization, significant increases in these antibodies were observed (Fig 4A). Priming with 50 μg of GP63-VLP elicited IgG induction similar to that of the 10 μg GP63-VLP group. However, mice receiving 50 μg of GP63-VLP displayed substantially higher antibody response upon boost immunization than the 10 μg group. Antibody responses evoked against the GP63 VLPs were similar to those elicited against the sonicated promastigote antigen. As expected, boost immunization resulted in a drastic increase in VLP-specific antibody response (Fig 4B).

### Increased frequency of B cells and GC B T cells in spleen

**Fig 5:**
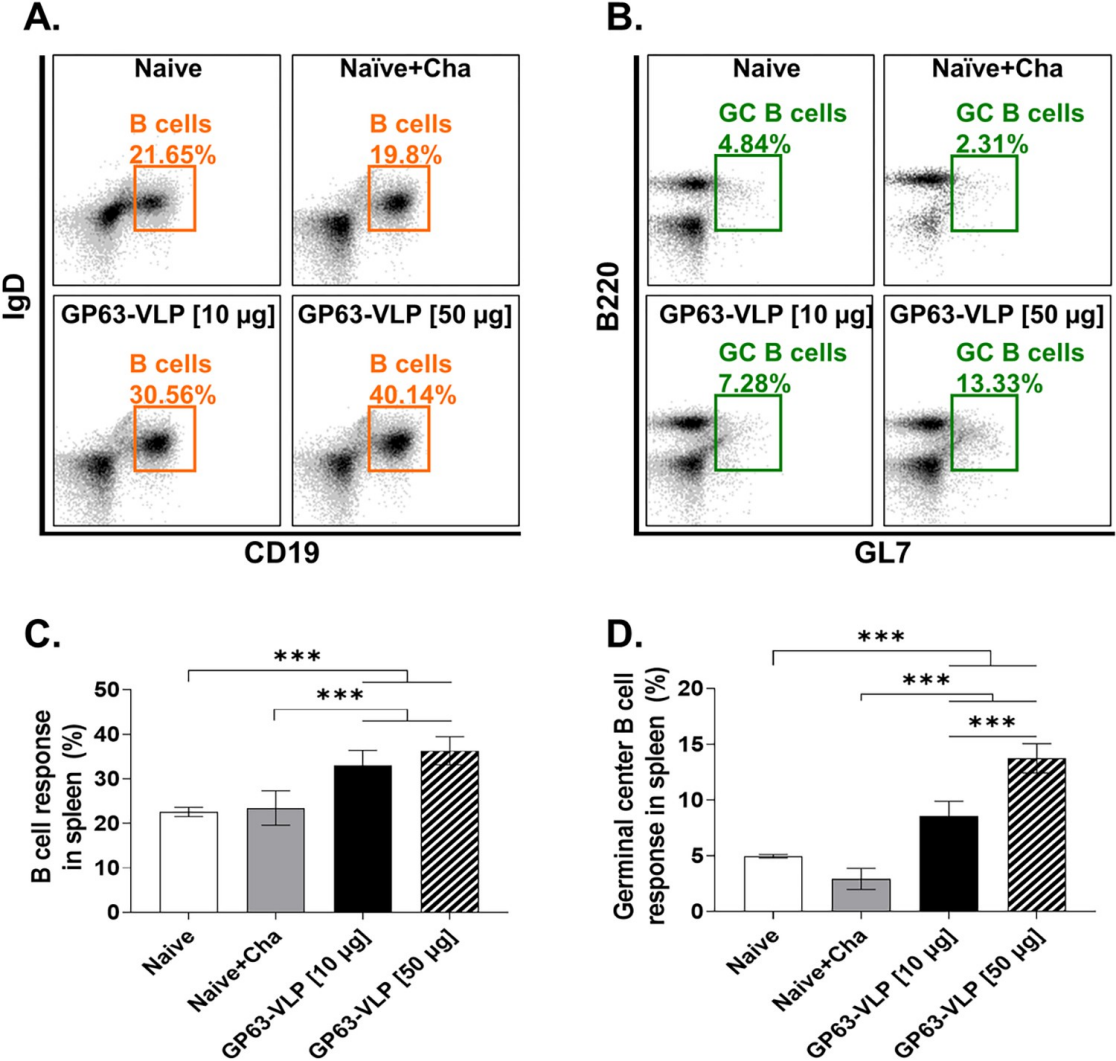
Vaccine-induced splenic B cell and GC B cell responses. Splenocytes were harvested and stained with respective surface markers for analysis using flow cytometry. Cells were gated accordingly to identify B cell and GC B cell populations. Representative scatter plots for B cells (A) and GC B cells (B) of each group were provided. Group-wise comparisons for B cell (C) and GC B cell (D) percentages were shown. Data are presented as mean ± SD, using individually processed samples (n = 8 per group). Asterisks denote statistical significance between the means of groups (***P < 0.001).

Flow cytometry analyses were conducted to evaluate the proliferation of B and GC B cells in the spleens of BALB/c mice. Notably, compared to Naïve, immunization with GP63-VLP significantly increased the proportion of B cells in the spleen (Fig 5A). Further investigation revealed a dose-dependent response in the induction of B cells and their subsets. Upon GP63-VLP administration, a significant increase in B cell induction was observed compared to the Naive+Cha. Though 50 μg GP63-VLPs elicited marginally greater B cell responses than 10 μg, the differences between the two VLP doses were not statistically significant (Fig 5C). The trend of dose-dependent induction was also evident in GC B cells following *L*. *donovani* infection (Fig 5B). Compared to Naïve+Cha, vaccination with both 10 μg and 50 μg doses of GP63-VLP significantly enhanced GC B cell numbers, demonstrating the vaccine’s efficacy in stimulating an immune response against *L*. *donovani* (Fig 5D).

### Increased frequency of CD4^+^, CD8^+^ T cells in spleen

**Fig 6:**
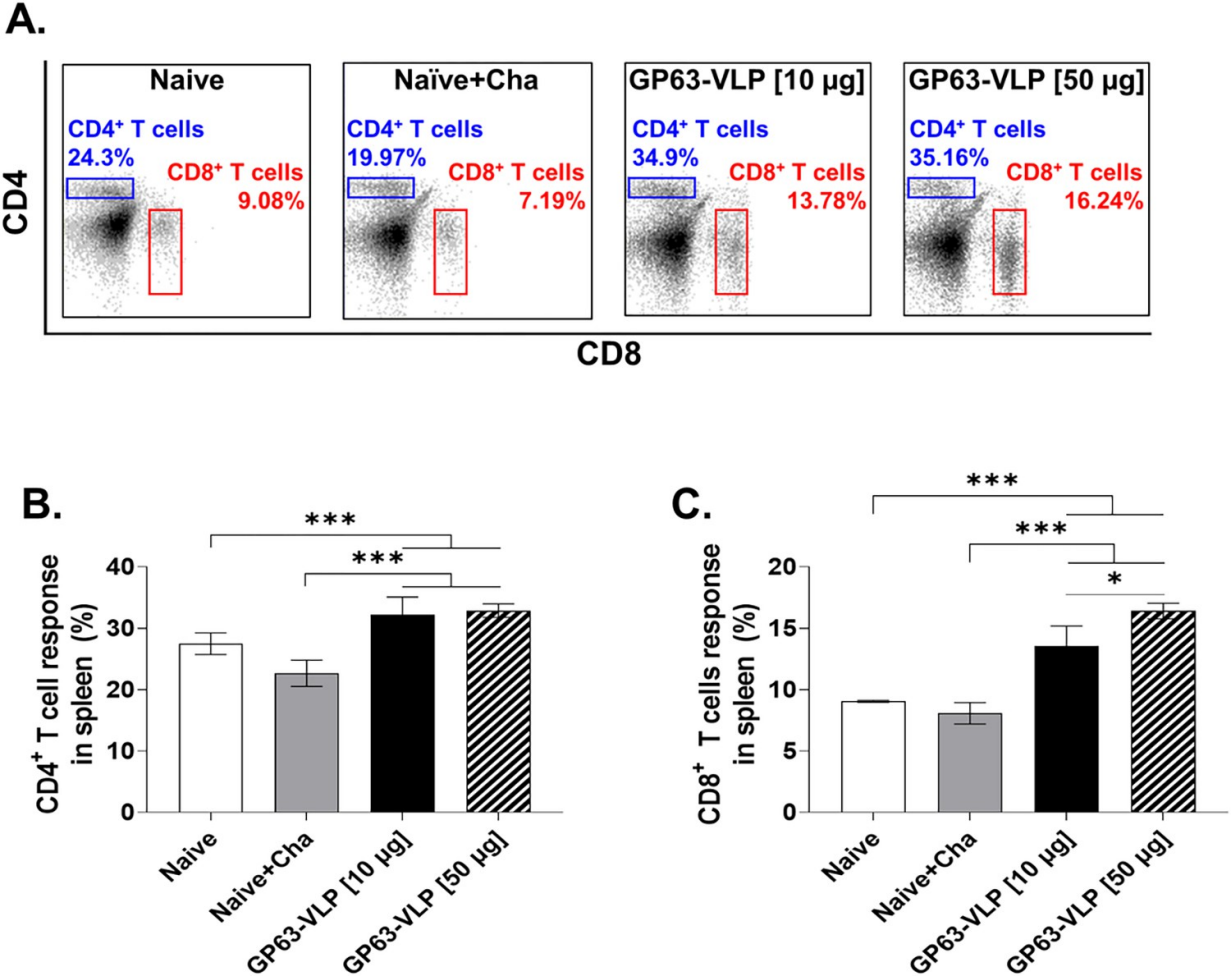
GP63-VLP vaccines increase the frequencies of splenic CD4^+^ and CD8^+^ T cells. Splenocytes were harvested and stained with respective surface markers for analysis using flow cytometry. Cells were gated accordingly to identify CD4 and CD8 T cells. Representative scatter plots and gating strategies were provided (A). Changes in splenic CD4^+^ (B) and CD8^+^ (C) T cell levels were assessed. Data are presented as mean ± SD, using individually processed samples (n = 8 per group). Asterisks denote statistical significance between the means of groups (*P < 0.05, ***P < 0.001).

Flow cytometry was performed to assess the proliferation of CD4^+^ and CD8^+^ T cells in the spleens of BALB/c mice. Specifically, immunization with GP63-VLP significantly increased the proportion of CD4^+^ and CD8^+^ T cells in the spleen compared to Naïve (Fig 6A). In contrast, the Naive+Cha showed a marginal reduction in both CD4^+^ and CD8^+^ T cells compared to the Naive. Upon administration of 10 μg or 50 μg of GP63-VLP, significantly higher levels of CD4^+^ T cells were observed compared to the Naive+Cha. However, this increase did not show dose-dependent statistical significance (Fig 6B). Vaccination with both 10 μg or 50 μg doses of GP63-VLP significantly enhanced CD8^+^ T cell proliferation compared to Naive+Cha, demonstrating the vaccine’s efficacy in stimulating immune responses against *L*. *donovani*. Notably, a significant increase in the T cell population was observed with 50 μg GP63-VLP compared to the other groups, indicating a strong immunogenic effect at this concentration (Fig 6C).

### Pro-inflammatory cytokine responses in the livers

**Fig 7:**
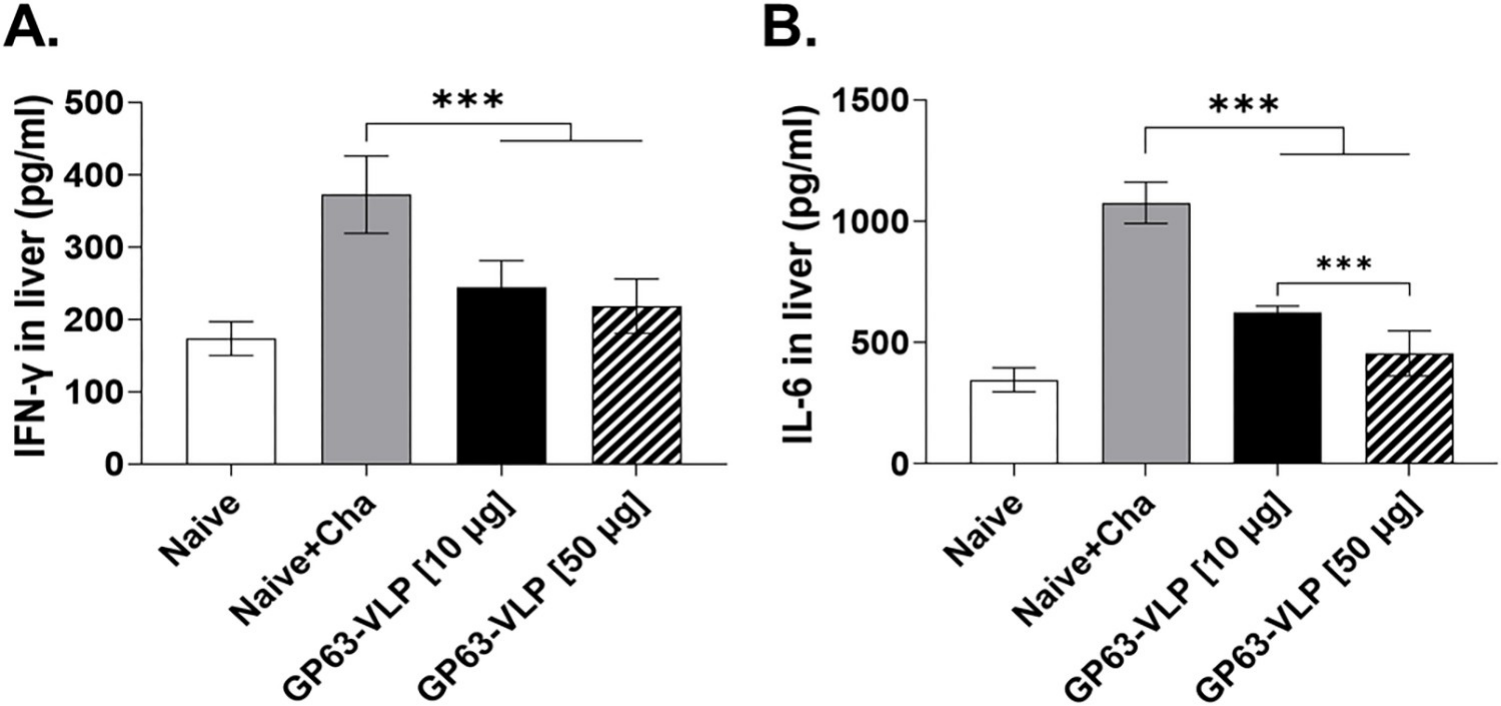
GP63-VLP vaccines inhibit inflammatory responses in the liver. Hepatic pro-inflammatory cytokine responses were measured. The presence of IFN-γ (A) and IL-6 (B) in the liver homogenates was quantified by cytokine ELISA assay. Cytokine concentration data are listed in S1 Table. Data are presented as the mean ± SD, using individually processed samples (n = 8 per group). Statistical significance between group mean values was indicated using asterisks (***P < 0.001).

Liver homogenates of mice were used to assess the production of pro-inflammatory cytokines IFN-γ and IL-6. In the Naive+Cha, *L*. *donovani* infection led to a substantial increase in hepatic IFN-γ levels. However, VLP immunization reduced the production of IFN-γ in a dose-dependent manner, with 1.52-fold and 1.7-fold reductions for 10 μg and 50 μg VLP doses, respectively, compared to the Naive+Cha (Fig 7A). Similarly, immunization with GP63-VLPs at 10 μg and 50 μg doses elicited approximately 1.72-fold and 2.36-fold decreases in the IL-6 response, respectively (Fig 7B). Overall, GP63-VLPs partially inhibited the production of inflammatory cytokines in non-lymphoid organs.

### Protective efficacy of GP63-VLP vaccines

**Fig 8:**
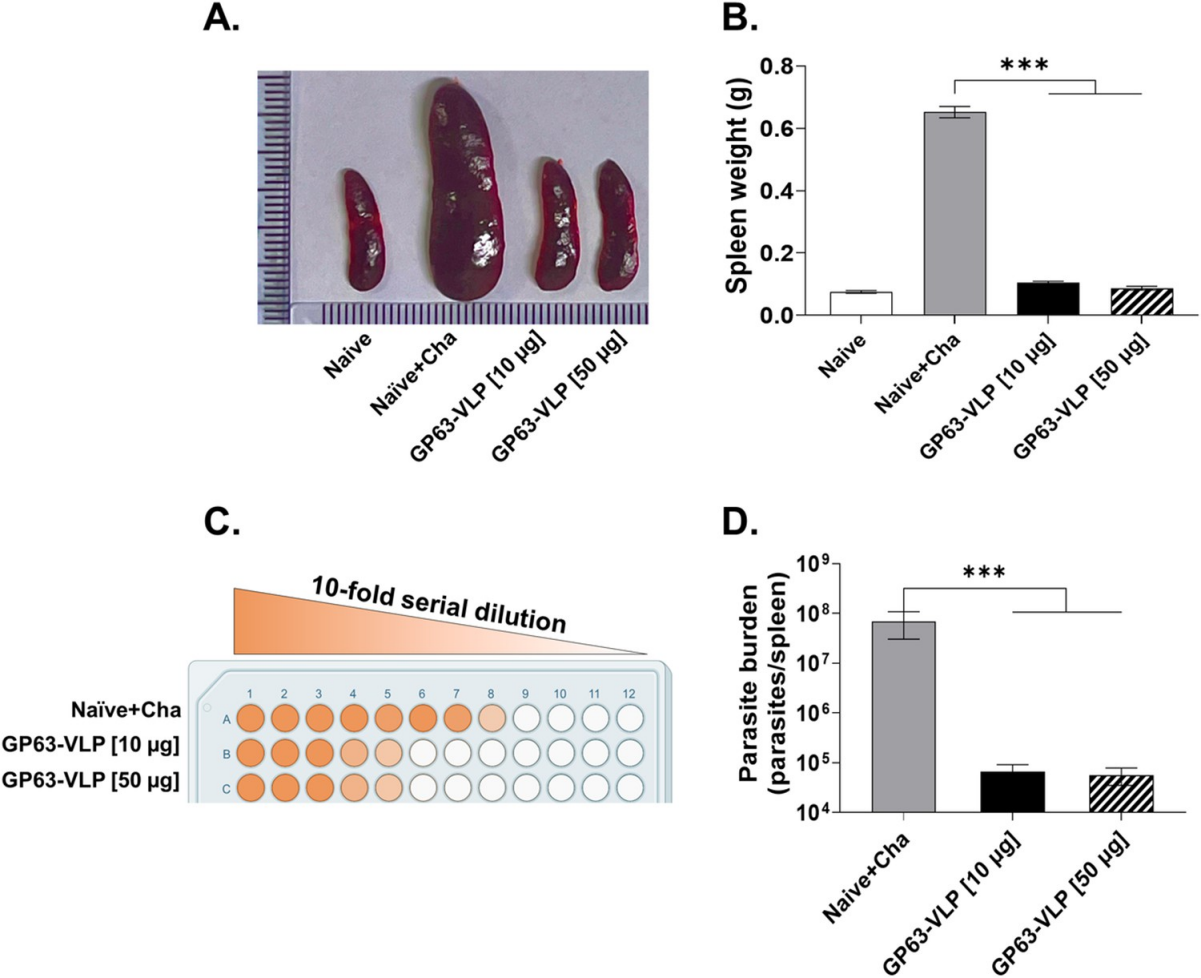
GP63-VLPs induce protection against visceral leishmaniasis in BALB/c mice. Vaccine-induced protection was evaluated by assessing the pathological changes elicited by *L*. *donovani* infection. Spleens were carefully harvested and the degree of splenomegaly was compared via size comparison (A). Individual spleens of mice were weighed and their overall weights were compared across all groups (B). A visual representation of the limiting dilution assay for parasite burden calculation was provided (C). Parasite burdens of individual mice were quantified and the mean burdens from each group were shown (D). Data are presented as mean ± SD, using individually processed samples (n = 8 per group). Asterisks denote statistical significance between the means of groups (***P < 0.001).

Splenomegaly, commonly observed in mice after parasitic infections, was observed in the Naïve+Cha, aligning with established observations. Immunization with GP63-VLP mitigated this parasite-induced splenomegaly in a dose-responsive manner. Notably, the spleen sizes in both the 10 μg and 50 μg GP63-VLP groups were very similar to that of the Naïve (Fig 8A). Conversely, the spleens of the Naive+Cha were noticeably different, with a profound increase in splenic mass. Spleens in the naive and vaccine groups weighed approximately 0.1 g, whereas the Naive+Cha spleens weighed approximately 0.6 g and were six times heavier than those in the GP63-VLP vaccine groups (Fig 8B). To further assess the vaccine’s protective efficacy against *L*. *donovani* promastigote infection, amastigotes were cultured and analyzed using the LDA method in 96-well plates. Significant differences in quantified parasite burdens were observed between the Naïve+Cha and GP63-VLPs groups (Fig 8C). Additionally, the number of promastigotes in the spleens of vaccinated mice decreased by approximately 1,000-fold and 1,200-fold in the 10 μg and 50 μg GP63-VLP vaccinated groups, respectively (Fig 8C).

## Discussion

VLPs possess multiple intrinsic properties that make their application more favorable than traditional vaccine platforms, whether it be immunogenicity or safety aspects. Live-attenuated vaccines, inactivated vaccines, and other traditional vaccine preparation methods are usually effective, albeit being only applicable to pathogens possessing a simple life cycle. Parasites, whose complex life cycle involves multiple hosts with interchanging asexual and sexual reproduction stages, these traditional vaccination methods are deemed less effective [27,28]. Based on this rationale, we chose to assess the efficacy of the VLP vaccine as it could lead to a more favorable outcome compared to some of the other previously researched vaccine platforms. The present study demonstrated that VLPs expressing the GP63 antigen of *L*. *donovani* can be an effective vaccination approach for VL. Our findings revealed that GP63-VLPs induced robust parasite-specific antibody responses and cellular immunity to aid in parasite clearance. Vaccine-induced immunity lessened the pro-inflammatory cytokine influx and parasite propagation in the visceral organs.

Germinal center B cells were heavily affected by VL upon challenge infection with *L*. *donovani*. This finding from unimmunized mice was also anticipated as microarchitecture disruptions in the splenic white pulp can lead to impaired formation of GC B cells [29]. VLP vaccine immunization ensured that B cell responses were maintained at significantly higher levels and prevented GC B cell depletion in mice. Yet, in our previous study using PSA-VLPs, GC B cell populations were substantially increased even in unimmunized mice following *L*. *donovani* challenge infection [22]. Factors accounting for this conflicting GC B cell results between the VLP vaccine studies remain largely unknown, but it is plausible that the administration route could have played a role. For example, one study demonstrated that infection homogeneity arising from the administration route differs. In support of this notion, compared to the endovenous injection of parasites, intraperitoneal injection into BALB/c mice resulted in a higher homogeneity of infection [30]. To ascertain whether this factor truly influenced the outcome of VLP-induced immune profiles, their impact should be investigated as it would shed more light on improving immunization regimens for VL vaccines.

Controlling VL in BALB/c mice requires *Leishmania*-specific CD4^+^ and CD8^+^ T cell responses [31]. In our previous VLP vaccine study, we mainly focused on investigating the protective role of vaccine-induced humoral immunity and did not take into account the function of T cells which are crucial for parasite clearance [22]. Here, our findings revealed that VLP immunization induced a marked increase in both parasite-specific CD4^+^ and CD8^+^ T cells. In contrast, unimmunized mice experienced splenic T cell depreciations. Data acquired from the naïve+challenge group is consistent with the clinical findings reporting impaired parasite-specific T cell responses in infected patients which are restored after cure [32]. Furthermore, consistent with our previous PSA-VLP study [22], GP63-VLP immunization inhibited splenomegaly and substantially inhibited parasite proliferation in the spleens. Given that both CD4^+^ and CD8^+^ T cell populations were retained at significantly higher levels than basal controls in the immunized mice even after challenge infection, this likely contributed to suppressing parasite propagation in mice.

It is widely accepted that BALB/c mice are susceptible to developing VL during the early stage of infection, but this later becomes easier to control and infections are chronically maintained at low levels [33,34]. For this reason, carefully selecting a visceral organ that best represents the actual parasite burden in infection models is important. Previously, Carrión et al [35] revealed that the parasite burden in the livers and bone marrows of *L*. *infantum*-infected BALB/c mice tend to decline around 28 days post-infection (dpi) and either become undetectable or maintained at low levels by 56 dpi. Contrastingly, the splenic parasite burden remains several orders of magnitude higher than those observed from the bone marrow and liver irrespective of the infection dose. This is consistent with the earlier report describing self-resolving VL in the liver while chronic infection persists in the spleen [36]. Based on these findings, we assessed the vaccine-induced reductions in splenic parasites. As expected, parasite burden in the unimmunized mice was determined to be at least 1000-fold higher than those observed from the VLP-immunized mice. This was also accompanied by the drastic reduction in splenomegaly.

In summary, VLP vaccines induced protection and contributed significantly to parasite clearance. Though protective, the protection demonstrated here by the GP63-VLPs was far from optimal. To ensure near-complete parasite clearance, improvements to immunogenicity are essential. Further studies for GP63-VLPs involving adjuvant incorporation, heterologous immunization strategy, and other changes to therapeutic regimen could yield fruitful results and advance VL vaccine development.

## Supporting information

S1 Table Analysis of pro-inflammatory cytokine production. (XLSX)
